# AMi: a GUI-based, open-source system for imaging samples in multi-well plates

**DOI:** 10.1107/S2053230X19009853

**Published:** 2019-08-06

**Authors:** Andrew Bohm

**Affiliations:** a Department of Developmental, Molecular, and Chemical Biology, Tufts University, 136 Harrison Avenue, Boston, MA 02111, USA

**Keywords:** AMi, *AMiGUI*, automated microscopy, multi-well plates, crystallography

## Abstract

Described here are instructions for building and using an inexpensive automated microscope (AMi) that has been specifically designed for viewing and imaging the contents of multi-well plates. The *X*, *Y*, *Z* translation stage is controlled through dedicated software (*AMiGUI*) that is being made freely available. Movements are controlled by an Arduino-based board running *grbl*, and the graphical user interface and image acquisition are controlled via a Raspberry Pi microcomputer running Python. Images can be written to the Raspberry Pi or to a remote disk. Plates with multiple sample wells at each row/column position are supported, and a script file for automated *z*-stack depth-of-field enhancement is written along with the images. The graphical user interface and real-time imaging also make it easy to manually inspect and capture images of individual samples.

## Abstract 2

Described here are instructions for building and using an inexpensive automated microscope that is driven by a dedicated graphical user interface. The system facilitates the automated imaging and manual inspection of multi-well plates.

## 1. Introduction   

Multi-well plates are used in a broad range of biological and clinical work, and automated systems for imaging samples within such plates are not uncommon in very well funded laboratories. For most academic laboratories, however, systems of this type are prohibitively expensive. A system for screening for protein crystals was described in 2007 (Brostromer *et al.*, 2007 ▸), but this required some specially fabricated parts and there was insufficient detail for users to build their own copy of the device. In recent years, the cost of 3D printers and computer numerical control (CNC) machines has decreased significantly. These devices are capable of making reproducible, submillimetre-scale movements over large areas. An open-source, automated microscope system based on delta geometry developed for 3D printers was described in 2016 (Wijnen *et al.*, 2016 ▸). A second open-source microscope stage for high-content screening (HCS) was described a year later (Schneidereit *et al.*, 2017 ▸). A third system, built by rearranging the parts of an inexpensive CNC machine, was designed specifically for imaging the results of protein crystallization trials (Bohm, 2018 ▸).

Described here is a significantly improved version of the third system mentioned above. The new system has been made more stable by altering the geometry and materials of the *Y* axis, and a graphical user interface (GUI) now makes the system much easier to use. Limit switches on the translation axes simplify the alignment process and alleviate the need for recalibration if power is lost. In contrast to the earlier system, which relied on a combination of command-line Python scripts and repurposed machining software to control the microscope movements, the new system is controlled via a dedicated GUI (named *AMiGUI*) that has been written specifically for imaging samples within multi-well plates, including those with multiple samples at each row/column index. *AMiGUI* is used to align the microscope, adjust the focus, control the lighting, manually inspect samples, take snapshots and automatically collect either single photos or *z*-stacked images of each sample. *AMiGUI* also writes a script that can can be run after the images have been collected for users who want digital depth-of-field enhancement.

The electronics of the AMi system have also been significantly improved. Whereas the camera in the earlier system wrote images to an SD card, which needed to be manually removed and transferred to a separate computer for processing and viewing, images in the new system can be written directly to a computer disk. Thus, they are much easier to access, process, view and archive. The new system also uses a different camera, lens and microscope mounting system, and the incorporation of a Raspberry Pi minicomputer has alleviated the need for an external laptop or desktop. Collectively, these modifications have made the new microscope more versatile, more user-friendly and even less costly to build than the original device.

The sections below provide an overview of the hardware and software components of the system. Detailed construction notes, files for 3D-printing the specialized parts, the *AMiGUI* source code and computer networking instructions are provided in the supporting information.

## 2. Hardware   

**Figure 1:**
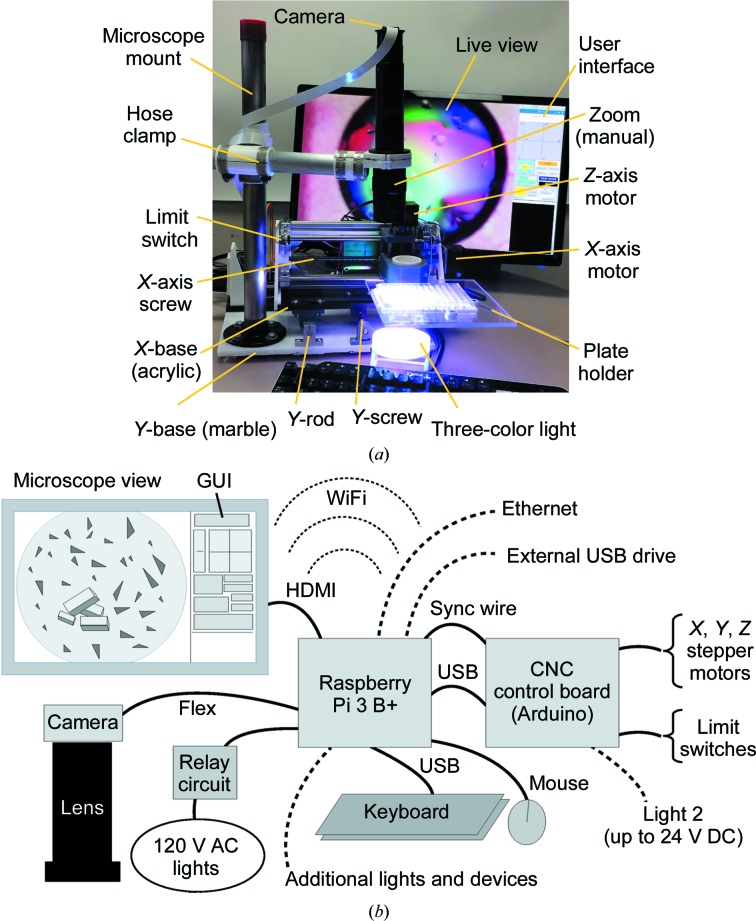
The automated microscope hardware. (*a*) Labeled image of the AMi system. (*b*) Wiring and connections between the component parts. Dotted lines indicate optional connections.

As in the earlier version of this microscope, the translation stage [Fig. 1 ▸(*a*)] is built around parts from a 1610 CNC machine. This machine is sold as a kit, and the kit contains the motors, the Arduino-based circuit board, the translation screws, the spring-loaded nuts and the other hardware necessary for precise, computer-controlled 3D movement. The new translation stage combines specially designed acrylic (or marble) pieces, 3D-printed parts, and aluminium and steel parts from the CNC machine kit. The *X* and *Z* translation assemblies are similar to those of the parent CNC machine. The *Y* translation assembly, however, differs significantly from both the manufacturer’s design and from the earlier version of the microscope. In the new system, the two metal rods that carry the weight of the system are secured to the base along with the translation screw and the *Y*-axis stepper motor. The bearings and spring-loaded nut are fixed to the bottom side of the acrylic piece that holds the *X* and *Z* axes.

Files detailing a 3D-printed sample holder for 96-well plates are included in the supporting information. While this new, 3D-printed sample holder is easier to fabricate, the earlier sample holder (constructed from a combination of 3D-printed parts and clear Plexiglas) is more flexible, as magnets above and below the Plexiglas can securely hold 12-, 24- and 96-well plates. All of these formats are supported by *AMiGUI*. Instructions for making both sample holders are included in the supporting information.

Two computer-controlled lights have been incorporated into the AMi system, and the angle and position of these lights can be adjusted manually while the image is displayed in real time. The lights are turned on and off via *AMiGUI*, which works though two general-purpose input–output (GPIO) pins on the Raspberry Pi. Users who wish to control additional lights or devices can do so using the remaining GPIO pins.

A three-colored light was constructed for examining protein crystals. This light source was inspired by the observation that a white light that illuminates only part of the field of view yields better contrast than one that illuminates the entire field (Supplementary Figs. S1 and S2). With the three-colored light, the red, blue and green LED modules each illuminate part of the field, providing enhanced contrast while illuminating the entire field of view. The distance between the diffusor (which was fabricated from 3D-printed white PLA plastic parts) and the colored LEDs is critical to the overall image contrast. While the three-colored light has clear advantages when imaging protein crystals, an offset white light yielded better images of cells, very small crystals and precipitates (Supplementary Fig. S3).

The microscope optics and camera are mounted above the translation stage, and their position is fixed during normal use; to adjust the focus, the sample is raised or lowered. As discussed in Bohm (2018 ▸), adjusting the focus in this way makes it easy to use a separate microscope with the automated translation stage and software described here. Alternative light sources are also easily accommodated. The AMi microscope mount is constructed from two pieces of metal pipe. The vertical segment is bolted to the base via a large flange, and the horizontal segment is attached to the vertical segment via a 3D-printed bracket. Another 3D-printed piece attaches the microscope lens to the end of the horizontal pipe. Pipe clamps securely hold the pieces of the microscope mount in place.

A Raspberry Pi 3 B+ single-board computer running Linux-based Raspbian is used to control the automated microscope system. This small, quad-core, 64-bit computer communicates with the Arduino board via a USB connection. Also connected to the Raspberry Pi are a USB keyboard and mouse, a 1080p monitor (connected via an HDMI cable) and an 8 megapixel picamera v.2.0 (connected via a 24-inch flex cable). Users may also choose to connect either an external disk drive or a removable thumb drive via the remaining USB port. Alternatively, as discussed below, images can be saved directly to a disk on another computer. The key connections between the various components are shown schematically in Fig. 1 ▸(*b*).

The picamera mentioned above is equipped with a tiny lens, which was carefully removed so that the image produced by the microscope falls directly onto the detector. A 3D-printed piece was designed to attach the picamera to the C-mount coupling on the microscope optic. Although the 8 megapixel picamera supports higher resolution modes, the 1640 × 1232 (2 megapixel) mode is arguably most useful. At this resolution, the system can display live video at 40 frames per second and files are written and transferred four times more quickly than is the case with 8 megapixel images. The 8 megapixel mode showed no significant improvement in image quality over the 2 megapixel mode; image quality appears to be limited primarily by the lens, not the detector. The picamera uses a ‘1/4’ detector (actual size 5.1 × 4.9 mm). The use of significantly larger detectors was discussed in the paper describing the earlier version of this microscope (Bohm, 2018 ▸).

Two microscope zoom lenses were compared: a 0.7–4.5× zoom lens from Thor labs ($1742 incuding a 1× extension tube and C-mount adapter) and an unbranded zoom lens with the same specifications from Amazon ($76, sold by aihome). The second lens included a 1× extension tube and did not require an adaptor. The unbranded lens lacks a focus ring, but this is not an issue since focus is adjusted by moving the sample closer or farther from the lens. While the image quality seems very similar, the depth of field is slightly larger for the more costly lens, and the images from the inexpensive lens have a small spot a little left of the center. All micrographs shown in the supplementary figures were taken using the inexpensive, unbranded lens.

## 3. Cost   

The cost of the component parts required to built the AMi system, including the lens, camera, monitor and control computer, come to roughly $500. The purchased parts are listed in Table 1 ▸.

## 4. Software   

**Figure 2:**
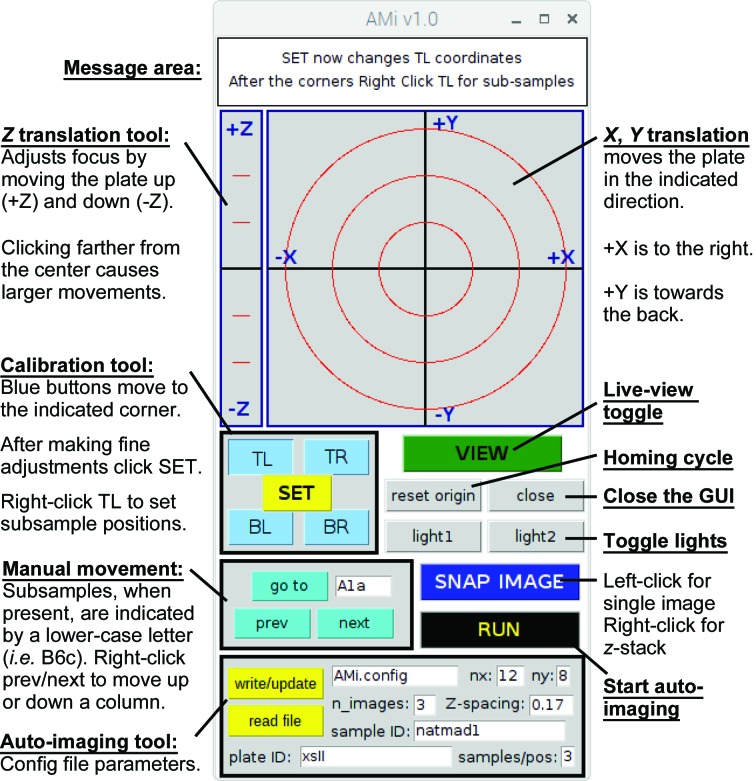
*AMiGUI* with brief descriptions of the key features.

The Arduino-based board that drives the stepper motors is running the CNC control software *grbl*, but AMi users will not interact with this directly except during the initial configuration. During normal use, the GUI, written in Python-Tk, runs on the Raspberry Pi and handles all communication with the Arduino board. The Python code includes extensive comments and is easy to modify. Successful startup of the GUI requires that a configuration file (AMi.config) be present in the directory from which the software is launched. This file contains the coordinates of the four corner samples, the number of rows and columns on the plate, and other information necessary for automated scanning. A sample AMi.config file is provided in the supporting information and the various parameters are discussed below. These parameters can be modified through the GUI (Fig. 2 ▸), and alternative configuration files can be read and/or written. Those scanning only one type of plate will need only one configuration file. Those scanning different types of plates will probably find it convenient to have a separate file for each type of multi-well plate.

To launch AMiGUI, the user types python3 AMiGUI.py. Once the GUI starts, users click ‘VIEW’ to open the live-view window. Buttons on the GUI control the lights. Both the live view and the lights are automatically turned off when automated imaging is complete. To facilitate alignment when creating a new configuration file or fine-tuning an existing one, the GUI has buttons that drive the sample to the coordinates (read from the configuration file) of each of the four corner drops. These are labeled ‘TR’ (top right), ‘TL’ (top left), ‘BR’ (bottom right) and ‘BL’ (bottom left). Manual adjustments to the *X* and *Y* positions are made using the part of the GUI that looks like a cross-hair. Clicking farther from the center results in exponentially larger movements in the direction indicated. A separate section of the GUI is used to translate the sample in *Z*, which changes the focus. Once the sample is centered and in focus, clicking ‘SET’ saves the coordinates of the corner that was most recently selected. During initial configuration, this process is repeated for each corner. It is very important that the corner samples be properly aligned and focused, as the coordinates of these samples are used to calculate, via bilinear interpolation, the positions of the intervening samples. The software can also be used to image plates with more than one sample well at each row and column (*i.e.* a plate with three small wells at each of the 96 positions). As discussed in the supporting information, this is performed by right-clicking the ‘TL’ button. The subwell calibration needs to occur after the positions of the four corner drops have been set.

AMi facilitates both manual inspection and automated imaging. Once the system has been calibrated, entering a row letter and column number (*i.e.* D5) will drive the microscope to the desired sample. The system also accepts numerical position input (*i.e.* entering 13 on a 96-well plate drives the system to position B1). Subwells are indicated by a lower-case letter (*i.e.* E3c or 63c). Buttons labeled ‘next’ and ‘prev’ are used to manually move from sample to sample. (*i.e.* …A11 > A12 > B1 > B2… or, if there are three subwells, …D3b > D3c > D4a > D4b…). Clicking ‘next’ or ‘prev’ with the right mouse button also moves the view up or down a row (*i.e.* D6 > E6). Left-clicking ‘SNAP IMAGE’ captures a single image that is named based on the sample position and the date (*i.e.* F3_Dec-25-2018-10:07AM.jpg). Right-clicking ‘SNAP IMAGE’ captures a series of *z*-stacked images where the number of images and the *z*-spacing between them is determined by the parameters entered into the lower part of the GUI. As discussed in the supporting information, a directory structure is used to keep the snapped images organized.

All image files are written to a directory named images. The program will stop with an error message if a directory with this name is not found. Although not recommended, the images directory can reside on the MicroSD card that also contains the microscope software and Raspberry Pi system. Those wishing to save images to an ext4-formatted thumb drive or a stand-alone USB hard drive can do so by creating a symbolic link named images that points to the desired location on the external device (*i.e.* ln -s /media/pi/USB-STICK/image_data images). The Raspberry Pi 3 B+ is equipped with both ethernet and WiFi, and the images can be transferred from the Raspberry Pi via SFTP (secure file-transfer protocol). It is even more convenient to to make a disk on another computer accessible via the NFS (network file server) protocol and then use a symbolic link to write the images directly to the remote computer as they are acquired. Instructions for doing this are provided in the supporting information.

Within *AMiGUI*, the ‘RUN’ button initiates automated imaging. At each sample position, a single image or a series of *z*-stacked images may be acquired. A separate directory structure is created for each sample and for each plate that is scanned, and the names of these directories are taken from the respective fields within the GUI. A subdirectory within each plate directory also holds any snapshots that have been taken. Additional subdirectories (named based on the date and time that the scan begins) are used to keep multiple scans of the same plate organized.

Imaging a 96-well plate with four *z*-stacked images of each sample takes about 12 min. The *z*-stacks are controlled by two parameters: the vertical distance between each successive image and the number of images in each stack. The optimal values depend on the thickness of the sample and the zoom setting (increased magnification reduces the depth of field). As discussed in the supporting information, the right-click snapshot function can be used to quickly test imaging parameters. Even with a very thin sample it may be advantageous to take more than one image because the calibration may be inaccurate, defects in the plate may cause the sample height to vary slightly or optical distortions owing to curved air–water interfaces may cause the focal point to shift slightly.

Once the images have been collected, the highest resolution features within each *z*-stack can be combined into a single picture with enhanced depth of field. This can be accomplished using the open-source program *Enfuse*, which is part of the *Hugin* package. Since this computationally intensive task is relatively slow (particularly on a Raspberry Pi), it is handled by a separate script which can be run either on the Raspberry Pi after all the plate scanning for the day is complete or on another Linux computer. A custom script for depth-of-field enhancement is written for each run. The images from a 96-well plate with four images per sample (384 images in total) take 48 min to process on the Raspberry Pi 3 B+. Processing the same 384 images takes 7 min on a 3.5 GHz Core I7 desktop.

## 5. Concluding comments   

The automated microscope described here is designed to accelerate the pace of research in laboratories that cannot afford an automated, commercial system with similar capabilities. Its low cost, physical stability, user-friendly interface, adaptability and the quality of its images should make the AMi system attractive to many researchers. This microscope could not have been built without powerful, inexpensive, open-source hardware and software. In keeping with this spirit, the detailed plans and the associated software are being made freely available.

Potential next steps on the software side include the creation of a dedicated software package for viewing and keeping notes on the collected images. Also, image-analysis software could be used to automatically center and focus the images and score the results of the screening experiments. On the hardware side, one might imagine the addition of a plate handler and a plate hotel. Alteration of the optics could also allow the system to function as a fluorescence or absorbed light plate scanner. Further modifications to the design and software will be posted to a website devoted to this project (https://abohm01.pages.tufts.edu). Users are strongly encouraged to share their experience with this device as well as hardware and software enhancements.

## Supplementary Material

Supplementary text and figures. DOI: 10.1107/S2053230X19009853/ft5102sup1.pdf

Technical drawings and files for 3D printing (zipped tar file). DOI: 10.1107/S2053230X19009853/ft5102sup2.zip

The Python program that controls the microscope. DOI: 10.1107/S2053230X19009853/ft5102sup3.txt

Sample configuration file for AMiGUI.py. . DOI: 10.1107/S2053230X19009853/ft5102sup4.txt

Video showing operation of AMi. DOI: 10.1107/S2053230X19009853/ft5102sup5.mp4

## Figures and Tables

**Table 1:** Component parts required to built the AMi system and their costs

Component	Cost (US$)	Source
1610 CNC machine kit	130	Ebay (various online sellers)
HDMI monitor	100	Micro Center
Microscope lens (0.7–4.5×)	76	Amazon (sold by aihome)
Raspberry Pi 3 B+	35	Adafruit
3.5 A, 5.1 V power supply	14	Amazon (sold by PWR+)
Picamera v.2.0	30	Adafruit
Keyboard and mouse	15	Micro Center
64 GB MicroSD card	12	Kingston
End switches (CYT1073)	9 (for 25)	Amazon (sold by Cylewt)
Pipe, flange and conduit six-pipe clamps, two-plug 120 V outlet	40	Home Depot
Marble, 1/2′′ thick, 1 × 2 feet	12	Home Depot
Colored 12 V LEDs: LE202-R013BLU12, LE202-R013RED12, LE202-R013GRE12	15	LED Supply
12 V DC power supply (2 A)	8	Amazon
Filament for 3D printing	20	3D Solutech
Two-channel 5 V DC relay module	8 (for three)	Amazon (sold by MCIGICM)
