# Supplementary material for: AMi: a GUI-based, open-source system for imaging samples in multi-well plates
Source: Acta Crystallogr F Struct Biol Commun. 2019 Aug 6;75(Pt 8):531–6. doi: 10.1107/S2053230X19009853 (PMC6688666; doi:10.1107/S2053230X19009853)
Supplement: Supplementary file 1 [file f-75-00531-sup1.pdf]

STRUCTURAL BIOLOGY
COMMUNICATIONS

Volume 75 (2019)

Supporting information for article:

AMi: a GUI-based, open-source system for imaging samples in multi-well plates

Andrew Bohm

White, diffuse light
from the bottom
(Low contrast in some
areas)

White, diffuse light
offset to the left.
(Great contrast, but
parts of the drop are
poorly illuminated.)

Tricolor light with the
Same geometry as the
top. (The whole
sample is illuminated
by three offset colors
and contrast is seen
between the colors.)

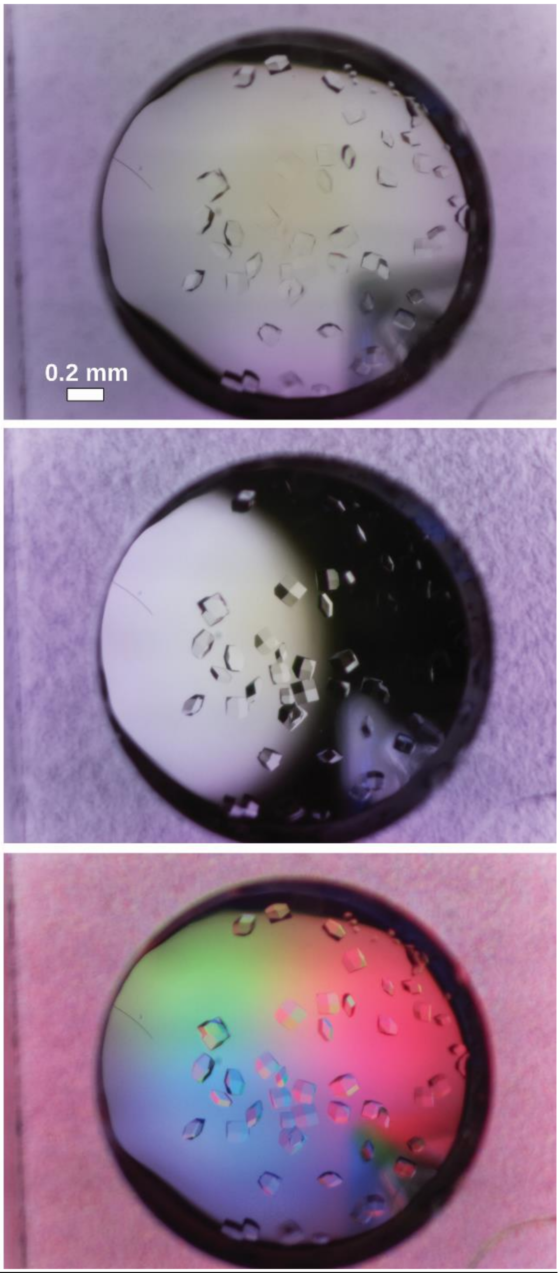

Figure S1 Imaging crystals with a 3-colored light source.

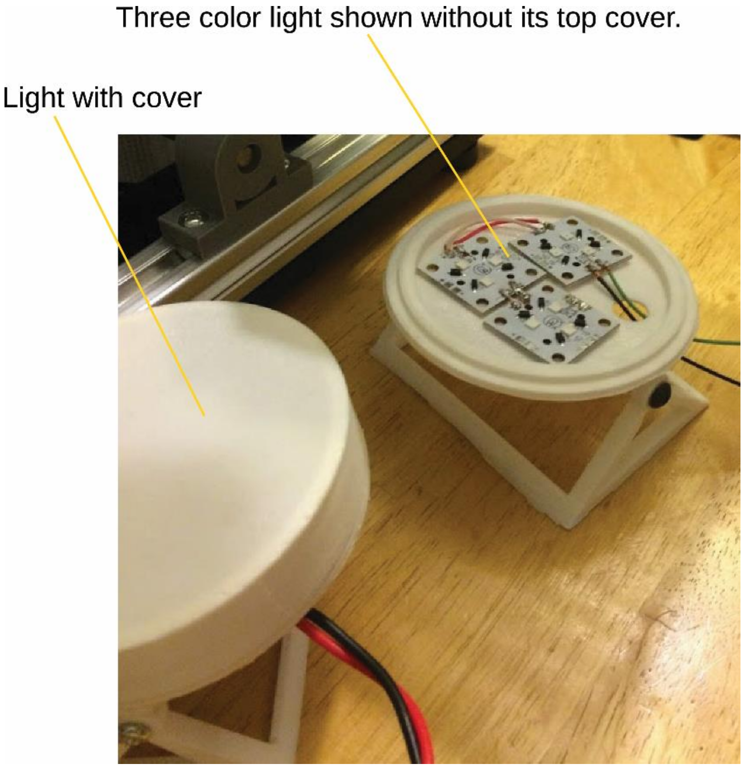

Figure S2 Construction of the 3-colored light source.

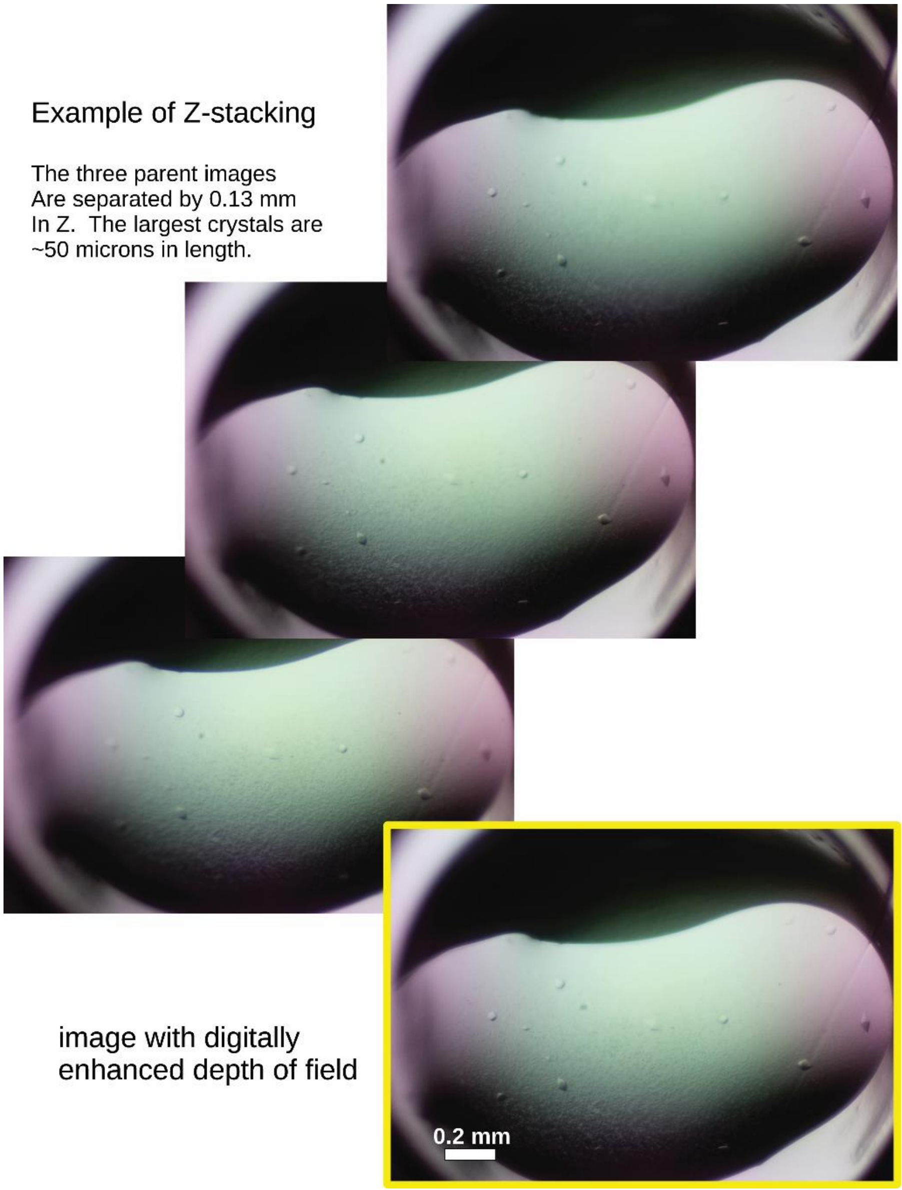

Figure S3 Sample composite image from a z-stack.

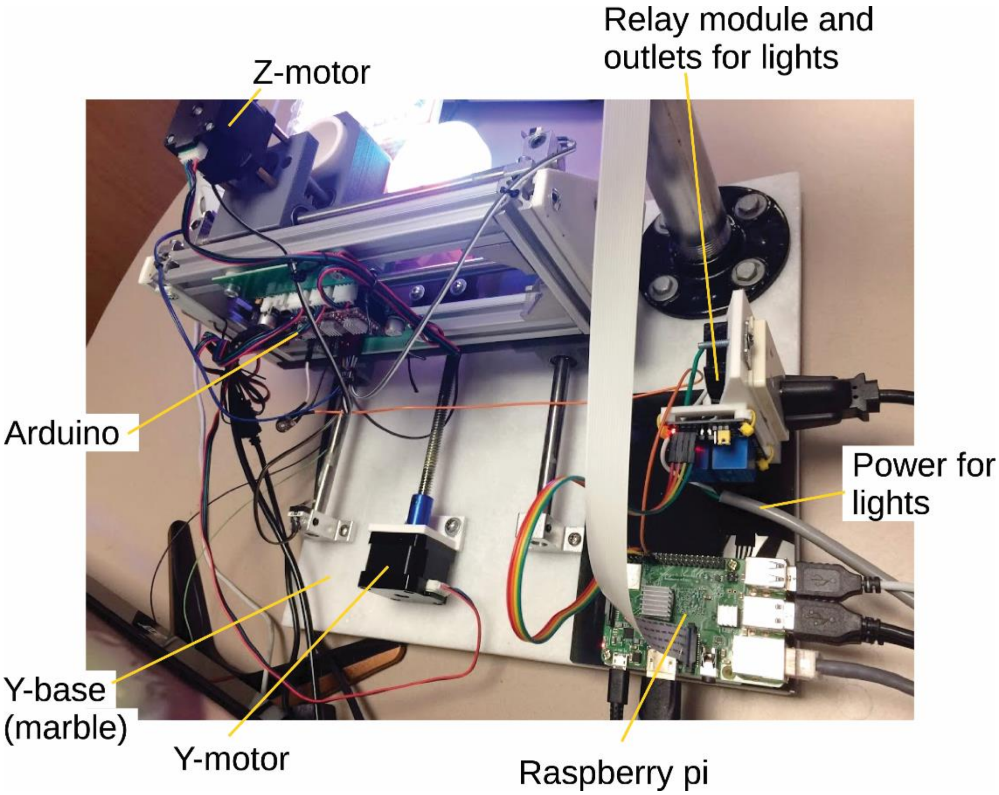

Figure S4 Rear view of the translation stage.

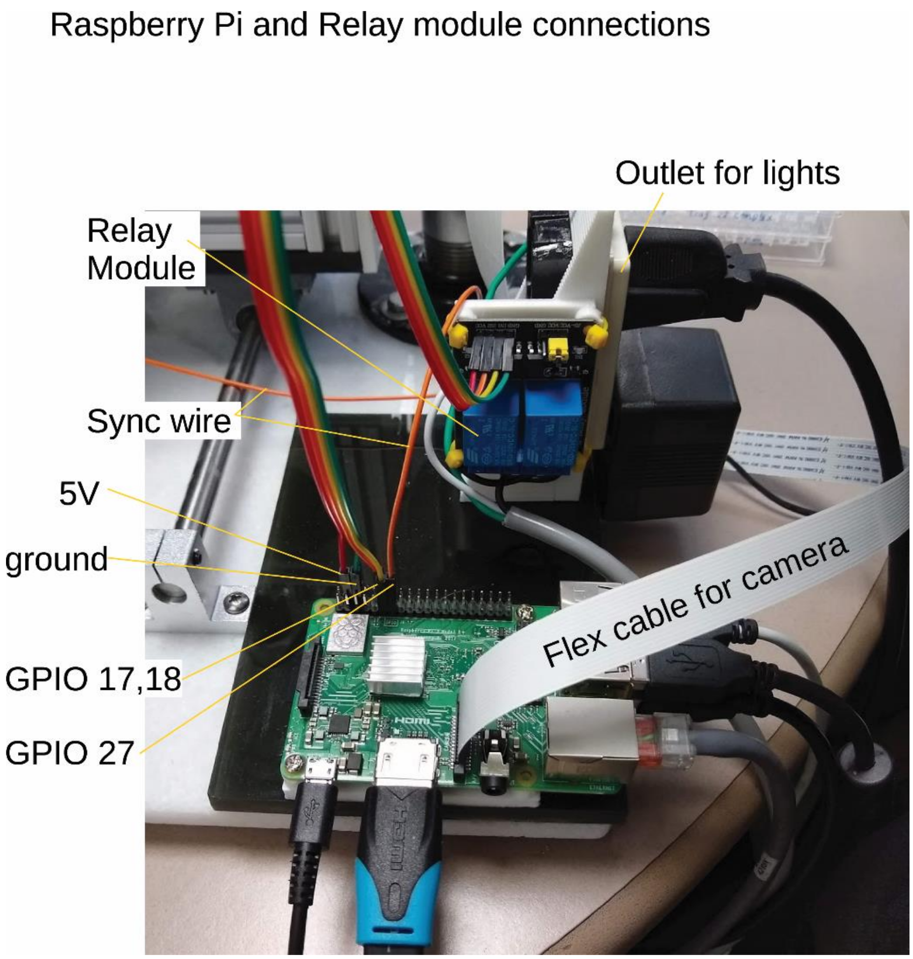

Figure S5 Closeup of the Raspberry Pi and wiring for the light switches.

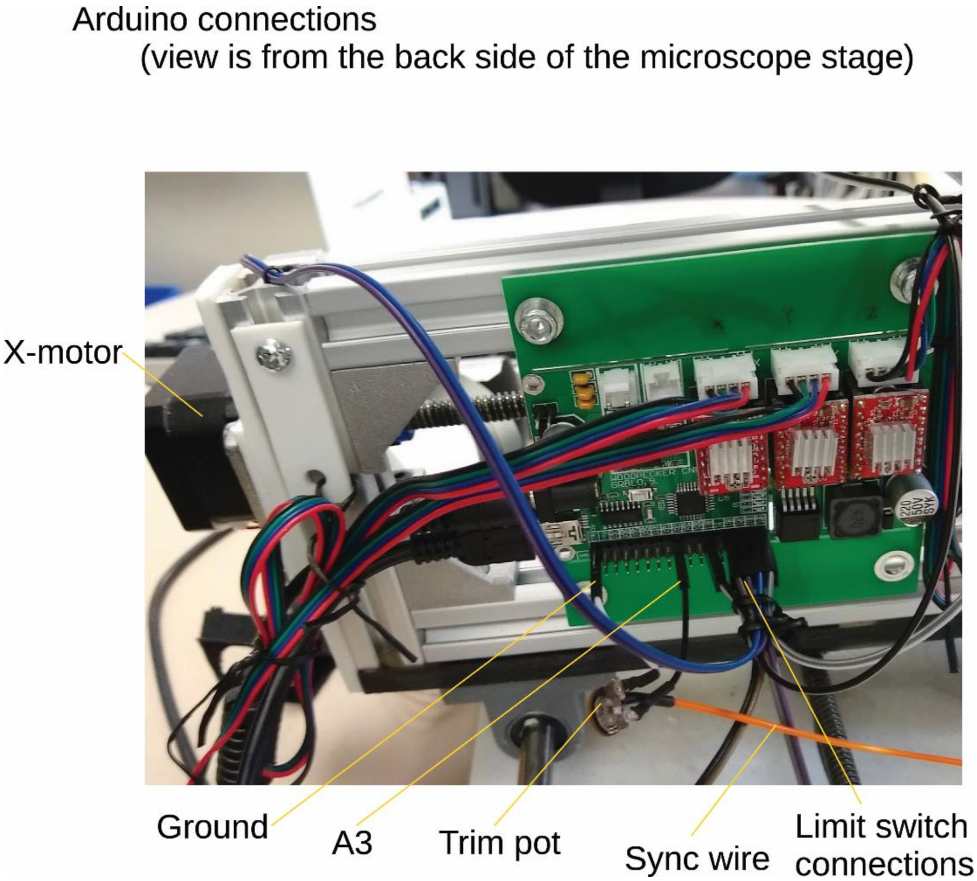

Figure S6 Closeup of the Arduino board.

S1. AMiGUI.py program

The AMiGUI.py program is included as a separate file. The program is written in Python3 and uses Tk to create the graphical user interface. As noted in the main text, the program will stop with an error if it does not find a control file named AMi.config and a directory or symbolic link named “images” in the directory from which the program is launched. To launch the code type `python3 AMiGUI.py`.

S2. Sample AMi.config file

```
12     8     1     # number of positions on x and y, then samples at each position
  140.776   26.256   15.856  # coordinates of the top left sample
   41.760   25.040   15.213  # coordinates of the top right sample
  140.008   89.497   17.232  # coordinates of the bottom left sample
   41.277   88.138   15.729  # coordinates of the bottom right sample
   0.0000   0.0000  # fractional offsets of sub-sample
    0.100 # zstep - the spacing in z between images
     3     # nimages - the number of images of each sample
lysozyme   # sample name
mcsg2_ab2     # plate_name
```

Please note that each additional sub-sample will cause one additional fractional offset line to be added to the file. The values in the file must be separated by at least one space. Also it is best to have the plate name be unique.

S3. Description of parts and assembly instructions

S3.1. Parts fabricated from flat material

X-base.fcstd (6 mm thick clear or colored acrylic)

overall dimensions are 260 mm x 90 mm

Y-base.fcstd (½ inch marble, wood, aluminum, or other stiff material)

overall dimensions are 410 mm x 305 mm

sample_base.fcstd (5mm thick clear acrylic)

overall dimensions are 175 mm x 185 mm

The measurements for each part are found in the supplied freecad (.fcstd) files. To maximize stability of the microscope stand, the large piece (Y-base) can be cut from a piece of ½ inch thick polished marble floor tile. Marble is recommended because it is very rigid and not expensive (about $12 for an 12x24 inch piece). Furthermore it is not much more difficult to cut and drill than acrylic or wood, though special drill bits and saw blades are strongly suggested. (To ensure the material does not crack,

drill and cut slowly, and be sure the material is well supported. A small amount of cooling water should be supplied during cutting and drilling.)

S3.2. Microscope mount

Parts: - 18” long segment of 1” diameter black pipe threaded on at least one side (you need ~38 cm)

Sanding the pipe will make adjustments easier, but this is not absolutely necessary.

- floor flange for 1” pipe
- 25 cm segment of 1” diameter electrical conduit

  (note that conduit is less heavy than black pipe and the diameter differs)
- 2-pipes.stl 3D-printed pieces
- big_lens_mount.stl 3D printed piece
- 6 hose clamps to secure the printed parts to the pipes

Tightly screw a 1” diameter length of black pipe into a floor flange. Then add the 90 degree connector piece (2-pipes), the second pipe, and the lens mount. The hose clamps are tightened only after the microscope is completely assembled. Note that the freecad files are also included. These can be edited (i.e. if a lens with a different diameter is used).

S3.3. Y-Base and Y-axis

Parts: - Y-base (made from marble or other material as described above)

- 4 metal rod holders (aluminium)
- Y_motor_mount.stl (3D-printed)
- Y-screw bearing
- 4 rubber stick-on feet (at least 10 mm high)

The Y-base is at the bottom of the translation stage. As noted above the Y-base piece can be made from laminated wood, acrylic, stone, or aluminum. Laminated wood (i.e. low cost shelving) is easiest to work, but depending on the laboratory environment, it may not last as long as other materials. Acrylic

is somewhat flexible, and aluminum is relatively expensive. Polished marble tile is ideal, since it is flat, stiff, water resistant, inexpensive, and readily available. Once the base is prepared, mount the four rod holders, the Y-motor mount (at the back) and the y-screw bearing (at the front). The screws should all be left loose at this point. The holes should all be larger than necessary so there is scope for minor adjustments. You can also screw on the microscope mount and then add the four feet. There is no need to install the two rods, and the other Y-translation hardware at this point.

S3.4. Preparation of the X-base and alignment of the Y axis

Parts: - X-base – acrylic piece described above

- one aluminum bar (220 mm)
- 4 bearings preinstalled in bearing mounts
- spring nut installed in its mount
- 2 rods (240 mm long) for the Y axis
- screw (240 mm long) also for the Y axis
- motor for the Y-axis
- motor-screw coupling piece

The X-base is above the Y-base, and travels forward and backwards along with the X and Z translation assemblies. The CNC kit includes screws with special heads that fit into the slot in the 220 mm aluminum bar. These screws are used to connect the bar to the back, top side of the X-base and the rear set of bearings. The acrylic X-base piece goes between the bar (on top) and the bearing holder (on the bottom). Bolts hold the assembly together on the bottom side, but there is no need to tighten them yet. With the rear set of bearings in place, screw the front set of bearings and the spring-nut assembly to the bottom side of the X-base. (The spring-nut assembly is attached to the middle of the X-base on the bottom side.) The front bearings and the spring nut assembly should be attached to the X-base using 3/4” (~20mm) screws. With these parts attached, add the lower set of rods for the Y-axis. These should

be inserted into the Y-axis rod-holders that are attached to the Y-base and the bearings attached to the X-base. By repeatedly tightening the various parts, testing the resistance by hand and making adjustments, find the position where the whole assembly moves along Y with minimal friction. Then add the screw, motor and motor coupling piece to the Y axis. Turn the screw by hand to move the X-base from one end of the Y axis to the other (this does not hurt the motor). Again, make adjustments as necessary so that the whole assembly moves with as little friction as possible. If there is significant resistance loosen the screws for the bearings and/or the spring-nut and then re-tighten. This is an iterative process, and it may take a few cycles of moving from one end to the other and loosening and tightening screws before the assembly moves smoothly over the entire range. When you are done, tighten everything well.

S3.5. X axis assembly

Parts: - left-side_90.stl, right-side_90.stl – 3D-printed parts that secure the ends of the X-axis.

- X-spacer.stl -spacer for the X-motor
- four rod ends
- one 220 mm aluminum bar (horizontal piece)
- two 115 mm aluminum bars (vertical pieces) These should be cut from longer bars.
- four corner brackets
- four rod holders (for the X-axis)
- Motor for the X-axis
- motor coupling piece

The X axis sits above the X-base. Create a rectangle from aluminum rods that builds up from the bar attached to the top, back side of the X-base. The short bars run from top to bottom, and the long bars run from side to side (inside the short bars). Secure the inside edges of all four corners using corner brackets. Screw the four rod holders onto the short aluminum bars, two on each side. There is no need

to tighten them yet. The X-axis uses the two triangular 3D-printed pieces (left-side_90 and right-side_90). The motor goes on the right side of the X-axis assembly and the X-spacer is used to translate it slightly to the right. Don't worry about the alignment yet. It is important that any protruding bits of filament be removed (with sandpaper) from the printed pieces before they are added. The printed pieces and the motor should all be screwed to the short rods.

S3.6. Adding the Z-axis assembly

Parts: - two 260 mm rods

- screw (260 mm)
- Z-axis assembly

The Z-axis assembly is preassembled in the CNC kit. To add it, run the two rods and the screw through the left side of the X-axis assembly and then through the Z-axis assembly. The motor on the Z-axis assembly should up at the top. You will probably need to fine tune the positions of the rod holders and the X-axis motor. As with the Y-axis, manually screw the X-axis from side to side making small adjustments until the translation is smooth throughout. Then tighten all the screws well.

S3.7. Mounting the circuit boards

Parts: pi-holder.stl 3D-printed piece

The two circuit boards should be mounted before you begin to make electrical connections. The Pi is attached to the pi_holder piece with zip ties and glued to the Y-base. The arduino is attached to the back side of the X-axis assembly with two screws.

S3.8. Sample holder

Parts: - 96_well_holder.stl (3D printed) only holds 96 well plates

- clear acrylic piece (sample_base.fcstd) 5mm thick 175 mm x 185 mm clear acrylic
- corner1.stl (3D-printed piece)
- two copies of corner2.stl (3D-printed)

- 6 neodynium magnets 10 mm in diameter
- plate_holder_cyl.stl (3D-printed)

The 96 well plate sample holder can simply be printed. The remaining directions are for making the adjustable sample holder (capable of holding plates of other formats). Screw the printed cylindrical piece to the clear acrylic piece and then glue with with acetone or superglue. The corner1 piece should be glued to the acrylic so it is flush to the left side and 56 mm from the back side. Glue (with epoxy) 3 magnets into each of the two 3D-printed corner2 pieces. Check the orientation of the magnets before adding the glue to be sure that the two finished corner2 pieces attract. These will be placed above and below the acrylic. When complete, the sample holder is installed by manually screwing the the Z-axis near the top of its translation and then pushing the cylindrical coupling all the way into the tool holder from the bottom side. The sample holder should be aligned by eye (the software will correct for misalignment) and then tightened in place with the screw on the z-axis assembly.

S3.9. Connecting the motors and switches

Parts: - 6 microswitches, wire, heat shrink

- z-block.stl (3D printed piece not needed on all systems)

Connect the motors to the Arduino following the manufacturers directions. Then connect the other devices as shown in Figure 1b. The end switches require careful assembly and planning. It is important to keep the switch wires physically separated from the motor wires as much as possible. Induced current can trigger an alarm and stop the machine. If the switch wires and the motor wires must touch, it is best that they cross at a 90 degree angle. Be sure to allow enough scope so that the wires are not stressed when the machine moves to its limits, but not so much that the wires can snag or get caught between the moving parts. Also, before you begin the solder wires to the switch terminals, you must identify the set of normally open terminals on the switch (so the circuit is closed when the switch is depressed). Carefully solder the wires to the six microswitches and cover the exposed ends

with heat shrink. The switches should be wired so that they are normally open. The switch wires should then be connected to the terminals labeled Xend, Yend, and Zend on the Arduino board. Some boards have only one Zend terminal. If this is the case wire the two Z switches in parallel and then connect them to the Zen terminal. The positive directions are up (for z), towards the back (for y), and toward the right (for x). The switches can be glued with Shoe-Goo to the rod holders on the x and y axes. Be sure to remove any grease from the surfaces before adding the adhesive. For the z-axis, the switches should be glued to the z-axis assembly with superglue. In all cases, the switches should be positioned such that the main body of the switch is flush with the edge of the piece it is being attached to. On some systems, the z axis translates to the point where the two grey plastic pieces come into contact. On others, there is a significant gap between the grey pieces at the end of the translation. In the latter case, the z-block can be used as a spacer. You will see the location of three switches and the z-block (white cube) in Figure 1a.

S3.10. Connecting the camera

Parts: - microscope lens

- picamera V2
- flex cable (24 inches long)
- sensor_holder_big_scope.stl 3D-printed part (should either printed in black or painted black)

Carefully remove the tiny lens on the pi camera. The lens typically has two tiny drops of sticky adhesive holding it in place. After removing as much of the adhesive as possible with a pin, carefully unscrew the lens while holding the camera with a small pair of pliers. Once the lens is off, install the microscope in the lens_holder (attached to the horizontal pipe) and the picamera in the sensor holder. Then connect the camera to the Raspberry Pi via the flex cable. Note that it is possible to focus the image even if the sensor holder is not precisely the correct length. Maintaining the focus when you change the zoom setting, however, requires that the sensor holder be precisely correct. If the focus is

lost when changing the zoom, try raising the sensor holder by a half millimeter. The sensor can also be brought closer to the lens by sanding a small amount of material from the lower end of the sensor holder. These fine adjustments can also be made after the entire system is complete.

S3.11. Light switch circuit and three-color light

Parts: - solenoid_holder.stl (3D printed part that holds the dual switch module)

- outlet_holder.stl (3D printed part that holds the outlet)
- light_parts.stl (3D printed parts for the light)

The two light circuits are controled by the GPIO ports 17 and 18 on the Raspberry Pi. The light 2 switch in AMiGUI also turns controls the 24 volt output of the Arduino board. The Raspberry Pi light circuits use an inexpensive two channel 5V DC relay module that is capable of switching both AC and DC current. Four wires connect the relay module to the Raspberry Pi: ground, 5V DC power, GPIO 17, and GPIO 18). A standard two plug, three-prong outlet was purchased, and the copper tab that separates the hot side of the two outlets was removed from the outlet. Using a standard 3-prong plug and wire, connect the ground and neutral wires to the outlet. Now connect the 120 V AC wire (with the wire unplugged of course!) to the two inputs on the high voltage side of the relay. Now connect the two normally closed, switched high voltage outputs to the two power terminals on the outlet. A 3D printed piece was designed to hold the outlet. This should be glued to the Y-base along with the 2-relay circuit. Connected in this way the lights will turn on as soon as the system is powered up. The light can be turned on and off via AMiGUI, and they will stay off if AMiGUI is closed. The lights will come back on, however, if the Raspberry Pi is shut down. This is to remind users to turn off the power to the whole system after the pi is shut down.

The three-colored light is made from three 12V LEDs which are wired in parallel to the small 12V power supply. These are mounted within a 3D printed housing. The light assembly must be printed from white PLA. The thin top of the assembly diffuses the light. The top parts of the light and

the LEDs can be affixed with superglue. Screws attach the base to the round top section and allow adjustment of the light angle. As noted in the main text, while the colored light is somewhat better for examining crystals (the facets capture light from different angles), precipitates and cells are best viewed with an off-center white light. Having two computer-controlled light circuits allows one to quickly switch from one light source to another.

S3.12. Software installation

If you did not purchase a microSD card with the raspian operating system pre-loaded, the system can be downloaded from https://www.raspberrypi.org/downloads/. You will need the system with the desktop included. Once the microSD card has been installed in the raspberry pi and the pi has been powered up, copy the software (AMiGUI.py) and the configuration file (AMi.config) to the pi. This can be done via web browser or via a USB thumb drive.

When you start the system for the first time, your display will most likely have a black border around the edge. To use the entire display, edit `/boot/config.txt` and uncomment the line that says `disable_overscan=1`. Also, go to Preferences > Raspberry Pi configuration > Interfaces and click the circles to enable SSH and the Camera.

S3.13. Arduino-Pi timing connection

The Arduino and Raspberry Pi are connected via a USB cable. In addition, a separate wire is used to ensure that the two boards remain in sync during image acquisition. GPIO pins on the Raspberry Pi accept 3.3 volt signals. The header connection labeled A3 on the Arduino board puts out a voltage closer to 5 volts. Both boards share a common ground. A 1 Kohm trim pot can be used as a voltage divider to adjust the output of pin A3 to 3.3 volts. The two outer pins of the trim pot should be connected to A3 and ground on the Arduino. Then using picocom (`apt-get install picocom`) to communicate with the Arduino, pin A3 should be energized.

`picocom /dev/ttyUSB0 -b 115200 -l` (connects to the Arduino board)

`$X` (allows manual control)

`m8` (energizes pin A3)

`m9` (de-energizes pin A3 – but you don’t need to do this now)

Once the Arduino A3 pin is powered, the resistance of the trim pot output (usually the middle pin) can be adjusted so that the output pin reads 3.3V to ground. Then the center pin should be connected to the header corresponding to GPIO27 on the raspberry pi. The raspberry pi B+ pin layout can be found at https://www.raspberrypi.org.

S3.14. Images directory

As noted in the main text, image files will be written to a directory named “images.” This should reside in the same directory that contains the AMiGUI.py program. There are at least three options for storing the images.

1) The images can be stored on the SD card on the pi. Micro SD cards are less reliable than hard disks, particularly when subjected to extensive read/write cycles. Thus, saving images to the SD card is okay for testing, but not recommended for routine use.

To have the images written to the SD card, simply create a directory on the pi named images (`mkdir images`). In this case you will most likely use sftp to move the images to another location after they are collected.

2) The images can be stored on an external disk or thumb drive connected to the pi via USB. This avoids writing to the SD card and allows the images to be transferred manually to another computer.

Connect the external drive or USB stick - it should mount automatically. To find its location use the command df (`df`). You should see an entry for the external drive (i.e. /media/pi/USB_STICK). Now create a symbolic link named images that points to the external USB drive (i.e. `ln -s /media/pi/USB_STICK images`).

3) The images can be stored on a disk on another computer that is shared via NFS. This is the most convenient arrangement, but also a bit more complicated to set up. The directions below assume you have connected the Raspberry Pi via ethernet to a local network and that the computer with the external disk is running Linux. In the example, the Raspberry Pi has ip number 192.168.2.8 and the computer with the hard drive has ip number 192.168.2.18. This is just one of many possible configurations involving a shared disk. (Note that NFS can cause security issues if implemented on an unprotected network. To help mitigate this risk, users may choose to create a special user for the images.)

First export a directory on the disk you wish to share by adding a line like the one below to **/etc/exports** on the computer with the hard disk you wish to share:

```
/home/microscope/images 192.168.2.8 (rw,sync,no_root_squash)
```

Now issue the following commands on the computer with the hard disk:

```
sudo systemctl start nfs-kernel-server.service
sudo chmod 777 /home/microscope/images
sudo exportfs -a
```

Now set a static IP address on the rasperry pi by editing the file **/etc/dhcpcd.conf** and adding lines that define the interface, static IP address, router, and domain name server. For example:

```
interface eth0
static IP_address=192.168.2.8
static routers=192.168.2.1
noipv6
static domain_name_servers=130.64.215.166
```

Then create a symbolic link named images that points to the external disk.

```
sudo mount 192.168.2.18:/home/microscope/images /home/pi/images
```

If the Pi is turned off, and back on, you will need to issue the last line again to reestablish the connection to the remote disk. Adding this line to the end of **/etc/bash.bashrc** will cause it to be executed automatically whenever a terminal window is opened. Although it is technically the correct thing to do, it is best not to add the remote disk to the /etc/inittab because the Raspberry Pi will not boot if the remote disk is not found, and you will be locked out.

S3.15. Directory structure

AMiGUI will create and update a directory structure that is designed to help keep the thousands of images it collects organized by project, plate name, and date. The contents of this directory structure are summarized below, where tabs denote subdirectories.

`images` directory

    sample directory (i.e. `lysozyme`)

        plate directory (i.e. `mcsg1_ab3`) the name should be unique

            `snaps` directory (only created if there are manually takent snapshots)

                individual snapshots (i.e. `D6a_Mar-19-2019_02:1:32PM.jpg`)

                `process_snap.com` (only written if a z-stack of a single well is taken. This is done by right-clicking the snap button.)

            date directory (i.e. `Mar-22-2019_11:25AM`) autoimaging output images

                `rawimages` directory

                    individual images (i.e. `A1a_0.jpg, A1a_1.jpg, A1a_2.jpg…`)

                copy of the configuration file used for autoindexing (i.e. `AMi.config`)

                script file for processing the images (i.e. `process_mcsg1_ab3.com`)

                processed images (i.e. `A1a.tiff, A1b.tiff, A2a.tiff…`)

                    these are the final, digitally depth of field enhanced images.

S3.16. Grbl setup

The Arduino board uses the program Grbl to control the three stepper motors and respond to the limit switches. Before running AMiGUI, you need to configure Grbl using a text-based terminal. The arduino will remember the altered settings, so this only needs to be done once.

With the Arduino board connected to the Raspberry Pi, install picocom (`apt-get install picocom`). Then issue the following commands:

`picocom /dev/ttyUSB0 -b 115200 -l` (connects to the Arduino board)

`$21=1` (activates hard limits so the machine stops when switches are contacted)

`$22=1` (enables homing so the machine can find its zero position)g

`$3=7` (changes default direction, so + translations move towards the right, back, and top)

`$23=7` (sets zero at the left, front, bottom position)

`$$` (show current settings)

`$H` (test homing cylce – stage should move to the front left bottom position)

The `$H` command above is a test of the settings and switches. When you are done, just close the terminal window.

S3.17. Startup and calibration

To start the software, type `python3 AMiGUI.py`. You should get a welcome message on the terminal, the three axes should find their home position, and the GUI should open. It is essential that the positions where the four corner samples are centered and in focus be saved before automated imaging or manual viewing. The corner positions will be initially read from a configuration file (AMi`.config`), and the software with exit with an error message if this file is not present in the directory from which the software is launched. Those who use more than one type of multi-well plate will most likely want to create a separate configuration file for each type of plate. To read a different configuration file, enter the file name in the lower part of the GUI and then click “read config.” Even if an appropriate configuration file is present, it is generally a good idea to check the positions of at least one of the corner drops. Clicking the four blue buttons drives the stage to the selected corner. (If you are using a plate with more than one sample well at each position, center and focus on the top-right sample at each corner.) The precise X, Y position of the stage and the focus (Z) can then be adjusted using the top two

windows. Clicking farther from the center results in exponentially larger movements. Once a corner sample is centered and in focus click SET to save the position. Then move on to the next corner. To write the saved coordinates to the configuration file click “write config.” A configuration file will also be automatically written to the directory with the images when automated imaging is initiated.

An additional calibration step is necessary when using plates with more than one sample at each position (i.e. 96-well plates with three sub-wells at each of the 96 positions). The position of the sub-wells should not be entered until all four of the top-right corner wells have been set. To calibrate the sub-wells, the number of samples per position should be entered into the box at the lower right of the GUI. Values greater than 1 in this box alter the behavior of the blue TL button. Right-clicking the button allows you to set the position of the 2nd sub-well, right-clicking it twice sets the position of the 3rd sub-well, etc. There is no need to describe the position of the sub-wells at the other corners. When more than one sub-well is present, the image names will have a lower case lettered suffix (i.e. D11a, D11b, ...). The sub-well information is saved to the configuration file when you click “`save file`.”

S3.18. Manual inspection

As discussed in the main text, once the positions of the four corners (and sub-wells, if present) have been set, AMi can be used to manually inspect the plate. Left-clicking the Next and Prev buttons move the view forward or backwards. Right clicking these buttons moves the view up or down a row. The focus and position can be adjusted using the X,Y and Z windows, but these changes have no effect on subsequent Next or Prev operations. Clicking SNAP captures an image which is written to the directory specified by the plate_ID field in the lower part of the GUI. Snapped image files have a time-stamp within their name.

S3.19. Automated imaging

As discussed in the main text, automated imaging is initiated by clicking RUN. (If a run is started in error, click Stop/Close just once to stop the run. (Clicking twice will cause the program to

end completely.) The plate_ID field and the screen field in the GUI, as well as the date, determines the location of the image files, and a script for depth of field enhancement is written along with the jpg images. The critical parameters for automated imaging are the number of z-stacked images per sample and the distance in z between these images. To determine the depth of field (this gets smaller as the magnification increases) choose a small, well defined object in one of your wells. Then translate in z until it is just barely in focus. The terminal window reports the x,y,z coordinates (in mm every time you make a movement. Record the z value, when the object you chose is just beginning to go out of focus. Then move z in the opposite direction until the object is again just barely in focus and record z again. The difference between these is the depth of field. A similar procedure can be used to determine the depth of your drops. It is best to set the z-spacing about 20% smaller than the depth of field. Also, the total thickness of the image slices (z-spacing times n_images) should be at at least 30% greater than the thickness you determine optically. To test your chosen parameters right-click the SNAP IMAGE. This collects a series of images and writes a script file that combines them into a single z-stacked picture. Once the images in your stack are acceptable and the corners and sub-drops are all in focus, click RUN to initiate the auto-imaging. This procedure need only be done once, provided you do not reset the magnification, change lenses or change the volume of your drops.

Viewing the images

If you have chosen to take more than one image of each sample you will likely want to run the image enhancement script (i.e. `source processMCSG2.com`). Then view the images using a photo viewing application such as Ristretto Image Viewer. To view the images on a remote machine, first install ssh on the server with the images (`apt-get install ssh`). Then start the ssh service (`sudo service ssh start`). Then open the x-window enabled connection from the remote machine (`ssh -x pi@***.***.*.*`). The *s represent the IP number of the server.

S3.20. Troubleshooting

My automated imaging run starts fine, but then the images become increasingly off center.

Most likely, you accidentally centered on a non-corner drop when doing the calibration. Re-center the corner drops, and confirm that the microscope is actually imaging the corner drops by putting the tip of a pencil or some other pointy object into the field of view at each corner. Don't forget to save the configuration file after you've gotten the correct corners set.

It is also possible that one of the set screws connecting a motor to its screw has become lose. These can be tightened with a small Allen key. A drop of epoxy or locktite can be used to ensure that the screw does not come lose again.

There's nothing to focus on in one of the a corner drops.

Use the translation tool to move to a neighboring drop that does have something to focus on and push set. Then, center the corner drop in question and adjust X and Y and click set again. Alternatively, you can use an identical plate that does have something to focus on at the position in question. It may help to make a small mark with a magic marker.

I accidentally collected images with the wrong plate ID.

First, don't panic. All image directories have their own time stamp, so no data has been overwritten. It is not difficult to move the directory containing the data to the correct place. You can do this from the command line using the UNIX **`mv`** command. First change into the images directory

```
cd images
```

Then move the directory to the correct location

```
mv <wrong_sample_ID>/<wrong_plate_name>/<dir_name>
<correct_sample_ID>/<correct_plate_name>/.
```

The dot at the end of the line above is important. It means don't change the directory name.

How do I abort an automated imaging run once it has started?

Click the Stop/Close button in the AMiGUI window just once. It is normal for the program to capture a few more images before the run stops. Clicking Stop/Close during a run causes the run to stop but leaves the GUI running. At other times clicking this button causes the program to end completely. Clicking the button twice during a run will cause the run to stop and then AMiGUI will close. If the Stop/Close button does not work, you can also shut down the program by clicking the X in the upper right corner of the AMiGUI window.

AMiGUI won't start up and the machine is very close to one of the translation endpoints.

The program will not start up if an end-switch is activated. Turn the translation screws by hand to a position where no end switches are depressed. Then try restarting the software.

The machine stops and the software hangs for no apparent reason.

There are at least three possible causes:

1) It is possible that a screw or one of the bars are misaligned. To check this, turn the screws by hand. If you will feel significant resistance (particularly near the ends), this is probably the problem. If so, loosen the screws that hold the bar holders and/or the bearings in question, translate the screw by hand to the end that gives the problem, and then re-tighten the screw. It may be necessary to do this more than once.

2) Electrical interference could be causing one of the switches to register a momentarily closed state, thereby triggering an Alarm in the Grbl software. This is most likely to occur during movement, and the terminal window should show an Alarm message if this is the case. The solution is to make sure the switch wires are well separated from the current carrying wires.

3) It is possible that you have a bad SD card. The SD card can be tested by removing it from the Raspberry Pi and inserting it into another computer. These cards are inexpensive, and it is relatively easy to simply purchase a new card and then download and configure the software as discussed above.

The machine does not zero correctly at the start or when the reset button is clicked.

This is most likely an issue with a limit switch. Switches can be checked by unplugging them from the Arduino board and testing continuity with the switch open and depressed. The circuit should be closed when the switch is depressed.

The machine stops with a message about moving beyond its physical limits:

If you have extended the dimensions of the machine by using longer screws and bars, you may get software message saying that a physically-allowed move is not permitted. The software limits for the x, y, and z axes can easily be changed my editing the AMiGUI code. The relevant parameters are near the start of the python program.
